# Splice variants of the extracellular region of RON receptor tyrosine kinase in lung cancer cell lines identified by PCR and sequencing

**DOI:** 10.1186/s12885-017-3747-x

**Published:** 2017-11-09

**Authors:** Soundararajan Krishnaswamy, Abdul Khader Mohammed, Gyanendra Tripathi, Majed S. Alokail, Nasser M. Al-Daghri

**Affiliations:** 1 Biomarkers Research Program, Riyadh Biochemistry Department, College of Science, King Saud University, Riyadh, 11451 Saudi Arabia; 2 Prince Mutaib Chair for Biomarkers of Osteoporosis, Riyadh Biochemistry Department, College of Science, King Saud University, Box 2455, Riyadh, PO 11451 Saudi Arabia; 3 Sharjah Institute for Medical Research, University of Sharjah, Sharjah, United Arab Emirates; 4 Department of Biomedical Sciences, University of Westminster, W1W 6UW, London, UK

**Keywords:** RON, Macrophage stimulating protein, Alternative splicing, RON isoform, Receptor tyrosine kinase, Lung cancer

## Abstract

**Background:**

Altered expression of receptor tyrosine kinases (RTKs) is a major driver of growth and metastasis of cancers. Recepteur d’origine nantais (RON) receptor is a single-pass transmembrane RTK aberrantly expressed in a number of cancers. Efforts to block deregulated RON signaling in tumors using small molecule kinase inhibitors or antibodies are complicated by the presence of unknown number/types of isoforms of RON, which, despite having similar sequences, are localized differently and mediate varied functions. The objective of this study was to identify splice variants of RON transcripts between exons 1 and 10 that code for the extracellular region.

**Methods:**

Direct cDNA sequencing was performed for the transcript between exons 1–10 of *RON* by Sanger sequencing in various lung cancer cell lines.

**Results:**

PCR amplification and bi-directional sequencing of cDNA for section between exons 1 and 10 from lung cancer cell lines revealed the presence of several splice variants of RON transcripts; the variants were formed by skipping of exons 2, 2–3, 5–6, 6 and 8–9. Each of these transcript variants were found in one or more cell lines. While the variants formed by skipping of exons 2, 2–3 and 5–6 resulted in loss of 63, 106 and 109 amino acids, respectively, and didn’t cause reading-frameshift, the transcripts formed by skipping of exons 6 and 8–9 caused reading-frameshift. Splice variant lacking exons 8–9 was found in 13 out of 23 cell lines tested.

**Conclusion:**

Lung cancer cell lines contain several splice variants of RON which involve skipping of exons coding for extracellular region. Some of the splicing changes result in reading-frameshift and the N-terminally truncated isoforms are expected to be secreted out. The ubiquitous nature of alternative splicing events in RON suggests the need for isoform specific approaches to functional analysis and therapeutic targeting of RON.

**Electronic supplementary material:**

The online version of this article (10.1186/s12885-017-3747-x) contains supplementary material, which is available to authorized users.

## Background

RON is a member of the Met family of receptor tyrosine kinases (RTKs) and ample evidence suggests its deregulated expression and functioning in a number of cancers. RON is overexpressed in cancers involving organs such as skin, colon, breast, lung and kidney [1–8] and its overexpression has been shown to be associated with tumor aggressiveness and metastasis [9]. Knockdown of RON expression in different cancer cell lines using siRNA/shRNA suppressed tumorigenic properties [10–12]. Validation of overexpressed RON as therapeutic target, however, has been hampered by the production of several of its isoforms by tumors. Several alternatively spliced transcripts and their protein products have been described for RON in various cancer cell lines as well as in solid and pleural tumors [13]. Differential localization of RON isoforms has been confirmed by their presence in the tumor microenvironment, cytoplasm and nucleus [14–16]. Recently, Western blotting studies indicated the presence of several RON isoforms in each of several lung cancer cell lines, while many of these same cell lines did not contain wild type RON [15].

The alternatively spliced transcripts of RON, which are formed by skipping of one or more exons, and their isoform products, show considerable sequence similarity with each other and to the wild type, despite exhibiting diverse functionalities in cancers. The isoforms include both hyperactive [2, 8, 17] and N-terminally truncated dominant negative variants [13, 18–20]. Notably, ectopic expression of some of the RON splice variants in NIH3T3 cells lead to tumor formation in vivo [13].

Recently, we reported the presence of several, frequently occurring novel transcript variants involving exons coding for the intracellular region of RON in lung cancer cell lines [21, 22]; the current study was designed to identify the splicing variants that affect the extracellular region of RON using lung cancer cell lines.

## Methods

### Cell lines

SCLC cell lines H526 (CRL-5811), H446 (HTB-171), H249 (CRL-5827), H69 (HTB-119), H2171 (CRL-5929), H345 (HTB-180), H82 (HTB-175), H146 (HTB-173), H889 (CRL-5817) and H524 (CRL-5831), and NSCLC cell lines SW1573 (CRL-2170), H358 (CRL-5807), A549 (CCL-185EMT), H1838 (CRL-5899), H661 (HTB-183), H522 (CRL-5810), H1437 (CRL-5872), H2170 (CRL-5928), SW900 (HTB-59), H1993 (CRL-5909), SKLU-1 (HTB-57), H1703 (CRL-5889) and SKMES (HTB-58) were obtained from ATCC (Manassas, VA) and were cultured in RPMI 1640 medium (Gibco/BRL) supplemented with 10% (*v*/v) fetal bovine serum supplemented with L-glutamine and 1% (v/v) penicillin/streptomycin at 37 °C with 5% CO_2_.

### cDNA preparation, PCR and sequencing

Total RNA from the cell lines was isolated using TRIzol reagent (Invitrogen, Carlsbad, CA, USA) following manufacturer’s instructions. cDNA was synthesized using 1 μg of total RNA and oligo-dT primers by using Single Strand cDNA Synthesis Kit (Clontech, Palo Alto, CA,USA). cDNAs were PCR amplified using two pairs of RON specific PCR primers; first PCR reaction amplified 1187 nucleotides of RON reference mRNA sequence (NM_000247) with forward primer (located in exon 1) 5′-CAACTTGCCACTGAGCTGAGCATC-3′ and reverse primer (located in exon 7) 5′-CAATGGGACTGAGTGTCTGCTAGC-3′; the second PCR reaction amplified 789 nucleotides of RON reference mRNA sequence with forward primer (located in exon 6) 5′-GAGGAGTTTGAGTGTGAACTGGAG-3′ and reverse primer (located in exon 11) 5′-CACGTTGATACCCACACAGTCAGC-3′. PCR reactions were carried out using Phusion high fidelity DNA polymerase (New England Biolabs, MA). PCR conditions were: initial denaturation at 98 °C for 30 s followed by 30 cycles of i) denaturation at 98 °C for 10 s, ii) annealing at 60 °C for 20 s and iii) extension at 72 °C for 15 s. PCR products were treated with EXO-SAP-IT (USB, Cleveland, OH) to remove excess primers, following the manufacturer’s instructions and sequenced bi-directionally using the same PCR amplification primers. Sequencing was performed by employing Big Dye Terminator Chemistry (Applied Biosystems, Weiterstadt, Germany). Sequence deletions in the PCR products were identified by aligning sequencing chromatograms with reference RON sequence using Mutation Surveyor version 3.1 software (SoftGenetics, State College, PA). The nucleotide position numbering is relative to the first base of the translation initiation codon of the full-length RON coding sequence (CCDS 2807.1).

## Results

Several alternatively spliced forms of RON transcripts and their protein products have previously been reported. However, PCR amplification and sequencing of the entire coding sequence (4200 bps, consisting of 20 exons) might have failed to provide a complete picture of all the alternative splicing events due to the formation of multiple products, which are expected to be similar in sequence except for one or a few missing exons. Hence, we determined the splicing alterations involving exon(s) skipping by amplification of two short, overlapping sections of RON cDNA, which together code for the complete extracellular portion of wild type RON protein. Results are summarized in Table 1.

**Table 1:** Summary of splicing changes identified in RON transcripts of lung cancer cell lines

Skipping of Exon(s)	Loss of nucleotides (from – to)	Nucleotides lost (n)	Frameshift	Premature stop codon	Amino acids lost (n)	Freq. of occurrence (n/23)
2	1231–1419	189	No	No	63	1
2–3	1231–1548	318	No	No	106	4
5–6	1720–2046	327	No	No	109	1
6	1881–2046	166	Yes	Yes		6
8–9	2184–2439	256	Yes	Yes		13

**Fig. 1:**
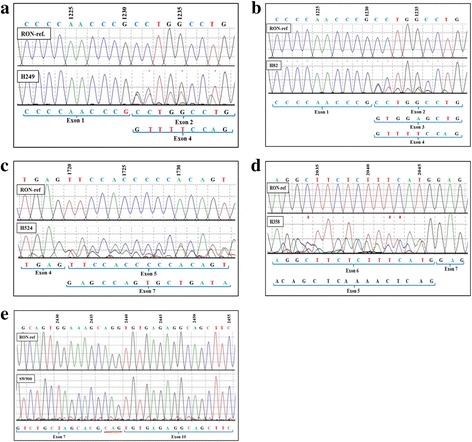
Identification of splice variants of RON transcripts in lung cancer cell lines by PCR amplification and sequencing of cDNAs. **a**) RON splice variant lacking exons 2 and 3 in cell line H249. Sequencing from 5′ end showed an additional overlapping sequence starting at nucleotide 1231 of RON reference sequence and the overlapping sequence corresponded to 5′ starting sequence of exon 4. **b**) RON splice variants lacking exon 2 and exons 2–3 in cell line H82. **c**) RON splice variant lacking exons 5 and 6 in cell line H524. This indicated the presence of a splicing variant caused by the combined loss of exons 5 and 6 and direct splicing of exon 4 with 7. **d**) RON splice variant lacking exon 6 in cell line H358. This indicated the presence of a splicing variant caused by loss of exon 6 and direct splicing of exon 5 with 7. **e**) RON splice variant caused by combined loss of exons 8 and 9 in cell line SW900. This indicated the presence of only the splicing variant resulting from the combined loss of exons 8 and 9 and direct splicing of exon 7 with 10

Sequencing from 5′ end of 1187 bps RON cDNA PCR products of cell lines indicated the presence of an overlapping sequence starting at nucleotide 1231 (immediately following exon 1) of RON reference sequence (Fig. 1A). The new sequence that appeared following exon 1 exactly matched with that of exon 4 of reference sequence suggesting the skipping of exons 2 + 3 in some of the RON transcripts. The exons 2 + 3 skipping splicing variant was found to be present together with the wild type RON transcript in cell lines H249, H69 and H526.

Additionally, sequencing from 5′ end of 1187 bps PCR products also showed the appearance of two overlapping sequences starting at nucleotide 1231 of RON reference sequence (Fig. 1B). Of the two overlapping sequences, one matched with that of exon 3 while the other matched with that of exon 4, suggesting the presence of two unique splicing alterations; one formed by skipping of exon 2 and the other formed by skipping of exons 2 + 3. H82 was the only cell line that contained both these splice variants along with the wild type transcript.

Sequencing the 1187 bps PCR products from the 5′ end also showed the appearance of an overlapping sequence starting at nucleotide 1720 of RON reference sequence (Fig. 1C). The newly appearing overlapping sequence matched with that exon 7 suggesting the presence of a splice variation involving skipping of exons 5 + 6, together with the wild type RON transcript. The exons 5 + 6 skipping splice variant was detected only in cell line H524.

In some cell lines, sequencing the 1187 bps PCR products from the 3′ end showed the appearance of an overlapping sequence starting at nucleotide 2046 of RON reference sequence (Fig. 1D). The overlapping sequence matched with that of exon 5 suggesting the presence of a splice variant formed by skipping of exon 6. RON splice variant formed by skipping of exon 6, which was present along with wild type RON transcript, was found in cell lines H358, H661, SW900, H522, H526 and H146.

Sequencing the 789 bps PCR products of lung cancer cell lines from 3′ end showed an abrupt change of sequence from nucleotide 2441 of RON reference sequence, and the new sequence matched with the 5′ sequence of exon 7 (Fig. 1E). This implied the presence of a splice variant resulting from the combined loss of exons 8 and 9 due to direct splicing of exon 7 with 10. Further, in addition to the direct splicing of exon 7 with 10, a codon CAG was also retained from the intron between exons 9 and 10. RON splice variant formed by skipping of exons 8–9, together with its wild type sequence, was found in cell lines H358, H661, H146, H524, A549, H1437, H2170, SKLU1, H249, SKMES, H69 and H522. However, in cell line SW900, normally spliced wild type RON variant was not found in cell line SW900, while exons 8 + 9 skipping variant was detected.

**Fig. 2:**
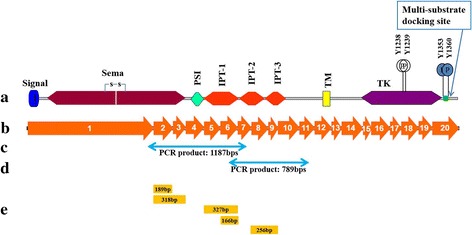
Schematic diagram of splice variants of extracellular coding region of RON. **a**) Two juxtaposed Y1238 and Y1239 in the kinase domain; phosphorylation sites Y1353 and Y1360 in the C-terminal docking site for multiple substrates with src homology 2 (SH2) domain; the other important motifs/domains are secretory peptide signal sequence, sema, plexin, semaphorin and integrin (PSI), Ig-like, plexins, transcription factors (IPT), transmembrane (TM) and tyrosine kinase (TK). **b**) 20 coding exons of RON shown in proportion to sequence length. **c**) 1187 bps PCR amplicon sequenced in the present study. **d**) 789 bps PCR amplicon sequenced in the present study. **e**) Specific, skipped exons by RON cDNA in lung cancer cell lines, as identified by PCR amplification and sequencing in the present study are: exon 2 (189 bps); exons 2 + 3 (318 bps); exons 5 + 6 (327 bps); exon 6 (166 bps) and exons 8 + 9 (256 bps)

The specific exons involved/skipped in alternative splicing are shown schematically in Fig. 2, in the context of full length structure of RON protein (showing constituent domains) and coding sequence of RON transcript (showing the constituent coding exons). Also, a supplementary table (Additional file 1: Table S1) is presented showing all the splicing variations of RON identified so far in our lab (including this study and previous two reports [21, 22]) using lung cancer cell lines.

## Discussion

Aberrant expression of RON in tumors involves frequent alternative splicing of transcripts leading to the formation of several isoform products which differ in their functionalities. However, high similarity among the structures of transcript variants or the protein isoforms poses problems in therapeutic target discovery and validation. In this study, we screened lung cancer cell lines for splice variants between exons 1 and 10 of RON transcripts through partial cDNA amplification by PCR and sequencing. Results revealed the presence of splice variants lacking exons 2, 2–3, 5–6, 6 and 8–9 in lung cancer cell lines. Of these, skipping of exons 2, 2–3 and 5–6 merely involved loss of 63, 106 and 109 amino acids, respectively, but did not cause frameshift; however, splice variants formed by skipping of exons 6 and 8–9 resulted in frame-shift together with the appearance of premature termination codons.

Alternative pre-mRNA splicing that eliminates exon 2 resulting in in-frame deletion of 63 amino acids in the extracellular domain of the RON β chain caused the production of a 165 kda variant that localized in the intracellular compartment [23]. Although it lacked tyrosine phosphorylation activity, the RONΔ165E2 variant was shown to phosphorylate and activate phosphatase and tensin homolog (PTEN). In the human colorectal cancer cell line HCT116, an in-frame deletion of 106 amino acids in the extracellular domain of RON β-chain caused by the deletion of exon 2 and exon 3 was shown to result in the production of a 160 kDa RON isoform, RONΔ160(E2E3), that localized intracellularly [24]. This isoform had no tyrosine phosphorylation ability, but it could constitutively activate Akt activity in transfected HEK293 epithelial cells. Splicing changes affecting the extracellular region have been shown to form dominant negative isoforms of RON, either secreted or intracellular, in cancers. Variant RONΔ85 is a soluble/secreted protein derived from a differentially spliced mRNA transcript arising out of retention of 49 nucleotide intron sequence between exons 5 and 6 [25]. RONΔ90 is another secreted RON isoform, coded by a transcript lacking exon 6 and as a result is N-terminally truncated [18]. Both these isoforms were detected in conditioned media in in vitro cell culture studies and were shown to be capable of binding both MSP and RON and block MSP/RON signaling. RON160 is a previously reported splice variant generated by deletion of 109 amino acids encoded by exons 5 and 6 [13, 20]. RON160 showed cell-transforming activities in cell focus formation and mediated anchorage-independent growth and RON160-mediated EMT was shown to be associated with increased motile/invasive activity [26]. In our study, splicing involving loss of exons 5–6 was only identified in 1 of 23 lung cancer cell lines indicating that this is not a common occurrence in lung cancer.

The splice variants formed by skipping of exon 6 and exons 8–9 result in reading frame-shift and were found in several cell lines examined in this study. RON transcripts with combined deletion of exons 8 and 9 have not been reported previously. Deletion of exons 8 + 9 result in the appearance of a premature termination codon; this may lead to the absence of the transmembrane sequence, which is coded by exon 12 of wild type RON; consequently, the N-terminally truncated peptide with most of the extracellular sequence of wild type RON may be secreted out. However, the transcript variants caused by loss of exons without concurrent frame-shift may lead to constitutively active or dominant negative activities. We speculate that the secreted RON isoforms may also bind and dimerize wild type RON located on the surface of tumor cells or on cells such as macrophages of the tumor microenvironment. MSP-RON signaling in macrophages suppresses inflammation by downregulating iNOS and COX-2 expression and increasing the production of the anti-inflammatory cytokine IL-10 [27–29]. Hence, the soluble RON isoforms circulating in blood may serve to increase inflammation in distant organs, an important hallmark of cancer, and thereby facilitating metastasis.

Results from the present and the two earlier studies (performed in our lab) [21, 22] on RON splicing changes involving skipping of exons using lung cancer cell lines has revealed that incidence of splice variants is common in lung cancer. RON transcripts in H82 cell line showed splice variations at 7 locations suggesting their possible occurrence in various combinations in full length transcripts. H249, SW900, H526, H69, H524, A549 and H358 were the other cell lines that contained 4 or more alternative splicing events. Also, a majority of the exons of RON that were analyzed in these studies were involved in alternative splicing. Furthermore, in agreement with the identification of multiple alternatively spliced RON transcripts, our previous work showed the presence of multiple RON isoforms of different sizes in several lung cancer cell lines as identified by Western blotting [15].

This study was mainly focused on evaluating the incidence of splicing changes which may interfere with the qualitative and quantitative outcomes of various methods currently employed to study the role of RON signaling in cancer. Amplification and sequencing of smaller regions of the coding cDNA sequence has proved powerful enough to reveal the presence of several alternatively spliced mRNA transcripts in lung cancer that may have been missed by other methods. The results of this and the other two previous studies [21, 22] strongly suggest that the presence of various alternatively spliced mRNA transcripts and their isoform products must be taken into consideration while correlating expression of wild type RON to its functions in lung cancer.

## Conclusions

Alternative splicing is a major general mechanism of genetic alteration used by cells to increase the repertoire and the functions of proteins and cancer cells stand to gain significantly by using this mechanism. Even though more than 90% of known transcripts are understood to undergo alternative splicing, this fact, which can interfere with both qualitative and quantitative methods, is not given due consideration in basic research or during target validation studies. Identification of several alternatively spliced variants together with the structural data from this study shed light on the frequent and ubiquitous nature of alternative splicing in RON in lung cancer and suggests that isoform specific quantification and functional determination may be an important prerequisite for understanding the deregulated RON signaling in cancers.

## Additional file

Additional file 1: Table S1. Summary of splice variations identified in lung cancer cell lines. (DOCX 14 kb)

## Abbreviations

MSP: Macrophage stimulating protein
NSCLC: Non-small cell lung cancer
RON: Recepteur d’origine nantais
RTK: Receptor tyrosine kinase
SCLC: Small cell lung cancer
TM: Transmembrane

## References

[1] Camp ER, Yang A, Gray MJ, Fan F, Hamilton SR, Evans DB, Hooper AT, Pereira DS, Hicklin DJ, Ellis LM (2007). Tyrosine kinase receptor RON in human pancreatic cancer: expression, function, and validation as a target. Cancer.

[2] Wang MH, Kurtz AL, Chen Y (2000). Identification of a novel splicing product of the RON receptor tyrosine kinase in human colorectal carcinoma cells. Carcinogenesis.

[3] Kretschmann KL, Eyob H, Buys SS, Welm AL (2010). The macrophage stimulating protein/Ron pathway as a potential therapeutic target to impede multiple mechanisms involved in breast cancer progression. Curr Drug Targets.

[4] Maggiora P, Marchio S, Stella MC, Giai M, Belfiore A, De Bortoli M, Di Renzo MF, Costantino A, Sismondi P, Comoglio PM (1998). Overexpression of the RON gene in human breast carcinoma. Oncogene.

[5] Gaudino G, Avantaggiato V, Follenzi A, Acampora D, Simeone A, Comoglio PM (1995). The proto-oncogene RON is involved in development of epithelial, bone and neuro-endocrine tissues. Oncogene.

[6] Sakamoto O, Iwama A, Amitani R, Takehara T, Yamaguchi N, Yamamoto T, Masuyama K, Yamanaka T, Ando M, Suda T (1997). Role of macrophage-stimulating protein and its receptor, RON tyrosine kinase, in ciliary motility. J Clin Invest.

[7] Wang MH, Dlugosz AA, Sun Y, Suda T, Skeel A, Leonard EJ (1996). Macrophage-stimulating protein induces proliferation and migration of murine keratinocytes. Exp Cell Res.

[8] Zhou D, Pan G, Zheng C, Zheng J, Yian L, Teng X (2008). Expression of the RON receptor tyrosine kinase and its association with gastric carcinoma versus normal gastric tissues. BMC Cancer.

[9] Thomas RM, Toney K, Fenoglio-Preiser C, Revelo-Penafiel MP, Hingorani SR, Tuveson DA, Waltz SE, Lowy AM (2007). The RON receptor tyrosine kinase mediates oncogenic phenotypes in pancreatic cancer cells and is increasingly expressed during pancreatic cancer progression. Cancer Res.

[10] Logan-Collins J, Thomas RM, Yu P, Jaquish D, Mose E, French R, Stuart W, McClaine R, Aronow B, Hoffman RM (2010). Silencing of RON receptor signaling promotes apoptosis and gemcitabine sensitivity in pancreatic cancers. Cancer Res.

[11] Wang J, Rajput A, Kan JL, Rose R, Liu XQ, Kuropatwinski K, Hauser J, Beko A, Dominquez I, Sharratt EA (2009). Knockdown of Ron kinase inhibits mutant phosphatidylinositol 3-kinase and reduces metastasis in human colon carcinoma. J Biol Chem.

[12] XM X, Wang D, Shen Q, Chen YQ, Wang MH (2004). RNA-mediated gene silencing of the RON receptor tyrosine kinase alters oncogenic phenotypes of human colorectal carcinoma cells. Oncogene.

[13] Lu Y, Yao HP, Wang MH (2007). Multiple variants of the RON receptor tyrosine kinase: biochemical properties, tumorigenic activities, and potential drug targets. Cancer Lett.

[14] Angeloni D, Danilkovitch-Miagkova A, Miagkov A, Leonard EJ, Lerman MI (2004). The soluble sema domain of the RON receptor inhibits macrophage-stimulating protein-induced receptor activation. J Biol Chem.

[15] Kanteti R, Krishnaswamy S, Catenacci D, Tan YH, EL-H E, Cervantes G, Husain AN, Tretiakova M, Vokes EE, Huet H (2012). Differential expression of RON in small and non-small cell lung cancers. Genes, chromosomes & cancer.

[16] Liu HS, Hsu PY, Lai MD, Chang HY, Ho CL, Cheng HL, Chen HT, Lin YJ, TJ W, Tzai TS (2010). An unusual function of RON receptor tyrosine kinase as a transcriptional regulator in cooperation with EGFR in human cancer cells. Carcinogenesis.

[17] Angeloni D, Danilkovitch-Miagkova A, Ivanova T, Braga E, Zabarovsky E, Lerman MI (2007). Hypermethylation of Ron proximal promoter associates with lack of full-length Ron and transcription of oncogenic short-Ron from an internal promoter. Oncogene.

[18] Eckerich C, Schulte A, Martens T, Zapf S, Westphal M, Lamszus K (2009). RON receptor tyrosine kinase in human gliomas: expression, function, and identification of a novel soluble splice variant. J Neurochem.

[19] Jin P, Zhang J, Sumariwalla PF, Ni I, Jorgensen B, Crawford D, Phillips S, Feldmann M, Shepard HM, Paleolog EM (2008). Novel splice variants derived from the receptor tyrosine kinase superfamily are potential therapeutics for rheumatoid arthritis. Arthritis Res Ther.

[20] Zhou YQ, He C, Chen YQ, Wang D, Wang MH (2003). Altered expression of the RON receptor tyrosine kinase in primary human colorectal adenocarcinomas: generation of different splicing RON variants and their oncogenic potential. Oncogene.

[21] Krishnaswamy S, Mohammed AK, Amer OE, Tripathi G, Alokail MS, Al-Daghri NM (2015). Recepteur d'Origine nantais (RON) tyrosine kinase splicing variants lacking exons 18 and 19 occur ubiquitously in lung cancer. Int J Clin Exp Med.

[22] Krishnaswamy S, Mohammed AK, Amer OE, Tripathi G, Alokail MS, Al-Daghri NM (2016). Novel splicing variants of recepteur d'origine nantais (RON) tyrosine kinase involving exons 15-19 in lung cancer. Lung Cancer.

[23] Ling Y, Kuang Y, Chen LL, Lao WF, Zhu YR, Wang LQ, Wang D (2017). A novel RON splice variant lacking exon 2 activates the PI3K/AKT pathway via PTEN phosphorylation in colorectal carcinoma cells. Oncotarget.

[24] Wang D, Lao WF, Kuang YY, Geng SM, Mo LJ, He C (2012). A novel variant of the RON receptor tyrosine kinase derived from colorectal carcinoma cells which lacks tyrosine phosphorylation but induces cell migration. Exp Cell Res.

[25] Ma Q, Zhang K, Yao HP, Zhou YQ, Padhye S, Wang MH (2010). Inhibition of MSP-RON signaling pathway in cancer cells by a novel soluble form of RON comprising the entire sema sequence. Int J Oncol.

[26] Ma Q, Zhang K, Guin S, Zhou YQ, Wang MH (2010). Deletion or insertion in the first immunoglobulin-plexin-transcription (IPT) domain differentially regulates expression and tumorigenic activities of RON receptor tyrosine kinase. Mol Cancer.

[27] Witte M, Huitema LF, Nieuwenhuis EE, Brugman S (2014). Deficiency in macrophage-stimulating protein results in spontaneous intestinal inflammation and increased susceptibility toward epithelial damage in zebrafish. Zebrafish.

[28] Wang MH, Zhou YQ, Chen YQ (2002). Macrophage-stimulating protein and RON receptor tyrosine kinase: potential regulators of macrophage inflammatory activities. Scand J Immunol.

[29] Morrison AC, Wilson CB, Ray M, Correll PH (2004). Macrophage-stimulating protein, the ligand for the stem cell-derived tyrosine kinase/RON receptor tyrosine kinase, inhibits IL-12 production by primary peritoneal macrophages stimulated with IFN-gamma and lipopolysaccharide. J Immunol.

