# Supplementary material for: Splice variants of the extracellular region of RON receptor tyrosine kinase in lung cancer cell lines identified by PCR and sequencing
Source: BMC Cancer. 2017 Nov 9;17:738. doi: 10.1186/s12885-017-3747-x (PMC5679369; doi:10.1186/s12885-017-3747-x)
Supplement: Additional file 1: Table S1. — Summary of splice variations identified in lung cancer cell lines. (DOCX 14 kb) [file 12885_2017_3747_MOESM1_ESM.docx]

**Supplementary Table 1.** Summary of splice variations identified in lung cancer cell lines.

| **Skipped exon(s)** | **Loss of nucleotides**  **(from - to)*** | **Nucleotides lost (n)** |  | **Lung cancer cell lines** |
| --- | --- | --- | --- | --- |
| 2 | 1231 - 1419 | 189 |  | H82 |
| 2+3 | 1231 - 1548 | 318 |  | H249; H69; H82; H526 |
| 5+6 | 1720 - 2046 | 327 |  | H524 |
| 6 | 1881 - 2046 | 166 |  | H358; H661; SW900; H522; H526; H146 |
| 8+9  16  16+17  16-19  15-19  18+19  19 | 2184 - 2439  3353 - 3534  3353 - 3644  3353 - 3947  3272 - 3947  3645 - 3947  3811 - 3947 | 256  182  292  595  676  303  137 |  | SW900; H358; H661; H146; H524; A549; H1437; H2170; SKLU1; H249; SKMES; H69; H522  H82; H1703  H82  H1703; A549; H2170; H1437; H1993; SKLU1; SKMES; H82  SW900  H249; H82; H526; H69; H524; SKMES; H1703; H1437; H1993; SKLU1; H358; A549  H249; H82; H526; H69; H524; SKMES; H1703; H1437; H1993; SKLU1; H358; A549 |

*The nucleotide positions numbering was done relative to the first base of the translational initiation codon of the full-length RON coding sequence (CCDS 2807.1).
